# Moderate pre-stroke physical activity has a protective effect on symptoms of depression in the post-acute phase after stroke

**DOI:** 10.1038/s41598-026-51679-5

**Published:** 2026-05-26

**Authors:** Moritz Hahner, Christine Meisinger, Inge Kirchberger, Michael Ertl, Markus Naumann, Jakob Linseisen, Timo Schmitz

**Affiliations:** 1 Institute for Medical Information Processing, Biometry, and Epidemiology, IBE, LMU Munich, Munich, Germany; 2 Pettenkofer School of Public Health, Munich, Germany; 3 Epidemiology, Medical Faculty, University of Augsburg, Augsburg, Germany; 4 Department of Neurology and Neurological Rehabilitation, District Hospital Guenzburg, Guenzburg, Germany; 5 Department of Neurology and Clinical Neurophysiology, University Hospital Augsburg, Augsburg, Germany

**Keywords:** Stroke, Physical activity, Depression, Anxiety, Diseases, Health care, Neurology, Neuroscience, Psychology, Psychology

## Abstract

Post-stroke depression (PSD) and anxiety (PSA) are common stroke-related complications that negatively impact recovery and quality of life. Physical activity (PA) is a modifiable factor that may influence these conditions. This study aimed to examine the association between pre-stroke PA and symptoms of depression and anxiety in post-stroke patients at 3 and 12 months after discharge. The analysis was based on 821 patients from the SCHANA study, recruited between 2018 and 2022 at the University Hospital Augsburg. PA was assessed using the International Physical Activity Questionnaire (IPAQ) and categorized into low, moderate, and high activity level groups. Depression and anxiety were evaluated using the Patient Health Questionnaire (PHQ-9) and the Generalized Anxiety Disorder scale (GAD-7), respectively. Multivariable linear regression models were applied. Moderate PA before stroke was associated with fewer depressive symptoms at 3 months post-stroke compared to low PA (β = -1.01, p = 0.007), but not after 12 months. High PA showed no significant association at either time point. Additionally, no significant association was observed for anxiety outcomes. A strong correlation was identified between symptoms of depression and anxiety at 3 months follow-up (r = 0.75, p < 0.001). Engaging in PA before stroke may contribute to better mental well-being during the subacute phase of stroke recovery. The association between depression and anxiety highlights the potential of lifestyle interventions to impact mental health outcomes post-stroke. Physicians should pay close attention to both inactive and highly active patients, as poor mental health can negatively affect recovery process.

**Supplementary Information:**

The online version contains supplementary material available at 10.1038/s41598-026-51679-5.

## Introduction

Stroke remains a leading cause of mortality and disability worldwide, yet it is highly preventable^1^. In 2021, 84% of the global stroke burden was attributed to modifiable risk factors, with a population attributable fraction (PAF) of physical inactivity accounting for the loss of 11.3% (95% CI: 1.8 to 34.9) of stroke disability-adjusted life years (DALYs)^2^. Beyond the immediate physical and neurological effects, stroke often leads to post-stroke depression (PSD) and anxiety (PSA), particularly within the first year after an acute stroke event. PSD affects approximately one-third of stroke survivors^3^, while PSA is reported in at least one-fifth of cases^4^, with some studies indicating even greater prevalence rates^5,6^. Both conditions are closely linked and contribute to reduced quality of life, greater disability, higher mortality, as well as an increased risk of recurrent stroke^7–10^. Depression and anxiety often co-occur, with anxiety being a common comorbidity in depressive disorders^11^. Recognizing this overlap, the 5th edition of the Diagnostic and Statistical Manual of Mental Disorders (DSM-5) introduced the ‘anxious distress’ specifier to identify individuals with major depressive disorder (MDD) who also experience anxiety symptoms. The bidirectional relationship between depression and anxiety complicates diagnosis and treatment, as it remains challenging to determine whether anxiety triggers depression or vice versa^11^. Depression is associated with several underlying mechanisms, such as dysregulation of the hypothalamic-pituitary-adrenal (HPA) axis, alterations in neurotransmitter function, and changes in proinflammatory cytokine levels^12^. Less is known about the neurobiology of anxiety, but it may share some common mechanism like dysfunction of the HPA axis with depression^13^. Furthermore, alterations in the limbic system as well as genetic factors are described in the context of anxiety disorders^13,14^. While the benefits of increasing physical activity (PA) after stroke for psychological recovery are well-documented^15,16^, the role of pre-stroke PA remains underexplored. Existing evidence suggests that higher levels of pre-stroke PA may reduce stroke severity^17–19^ and mortality^19,20^, improve recovery outcomes^20–24^, and contribute to a later onset of first-ever stroke^22^. However, its potential to reduce symptoms of PSD and PSA has been explored in only two studies, which suggest a protective effect against depressive symptoms but emphasize the need for further research to provide stronger evidence^25,26^. Several potential mechanisms mitigating this protective effect have been proposed: Neurotrophic factors, for example Brain-Derived Neurotrophic Factor (BDNF), are released due to PA and promote neuronal growth and recovery^27^. Furthermore, PA might also reduce neuroinflammation by maintaining the integrity of the blood-brain barrier^28^ and stimulating cerebral angiogenesis^29^. This study aims to address this gap by investigating the association between PA before stroke and symptoms of depression and anxiety after stroke. Using longitudinal data from a prospective single-center observational cohort study conducted in Augsburg, Germany, we aimed to analyzed whether pre-stroke PA influences mental health outcomes at 3 and 12 months after discharge.

## Methods

### Study design

The Stroke Cohort Augsburg (SCHANA) is a prospective, single-center observational cohort study conducted in collaboration between the Institute of Epidemiology at the University of Augsburg and the Department of Neurology and Clinical Neurophysiology at the University Hospital Augsburg. The study includes two phases of recruitment: SCHANA, which took place from September 2018 to November 2019 (*n* = 950), and SCHANA-2, conducted between January 2020 and May 2022 (*n* = 840), resulting in a total cohort of 1,790 patients. All participants were aged 18 years or older with confirmed ischemic or hemorrhagic stroke who were treated at the University Hospital Augsburg. Patients were excluded if they were unable to understand the consent form and answer the questions due to language barriers and no relatives were available to assist with translation.

Baseline data were collected during the hospital stay using standardized interviews conducted by trained study nurses. If a patient was unable to provide self-reported information, a proxy interview was performed (a close relative or friend takes the interview on behalf of the patient). Clinical data, including comorbidities, diagnostic results, laboratory parameters, treatments, and prescribed medications, were extracted from medical records, and blood samples were obtained during the hospital stay. Follow-up assessments were carried out via self-administered postal surveys at 3 and 12 months after discharge. To minimize loss to follow-up, participants who did not return the questionnaires were reminded by telephone.

All data collection procedures were conducted in accordance with the Declaration of Helsinki^30^. Ethical approval was granted by the ethics committee of the Ludwig-Maximilians-Universität München (Reference No. 18–196), and all participants or their legal representatives provided written informed consent. Further details about the SCHANA study are available in the published study protocol^31^.

### Survey data

Baseline data were collected using standardized questionnaires that included information on sociodemographic characteristics, social network, former stroke events, smoking behavior, physical activity, general health status, and symptoms of depression and anxiety. Follow-up questionnaires, conducted at 3 and 12 months post-stroke, reassessed symptoms of depression and anxiety.

Data on educational level, classified according to the International Standard Classification of Education 1997 (ISCED-97)^32^, was collected from the participants. The living situation was recorded and was used as an indicator of the participant’s social network in the present analysis. A variable was created to differentiate between participants living solitary and those cohabiting. Patients with recurrent stroke were identified based on self-reports and data extracted from medical records. Smoking status was assessed using an adaptation of the German National Cohort^33^ questionnaire and categorized into non-, former and current smokers. The EQ-5D VAS (EuroQol-5 Dimension Visual Analogue Scale) was used to assess patients’ overall health status on the date of questionnaire completion. It is a 0–100 scale, where 0 represents the worst imaginable subjective health status and 100 represents the best. This scale captures the patient’s perspective, providing a direct reflection of how they perceive their health at a given point in time^34^.

### Pre-stroke physical activity (exposure)

The level of PA seven days prior to the stroke event was assessed during the baseline survey using the short form of the International Physical Activity Questionnaire (IPAQ). Participants reported the spent with any form of PA, like walking or engaging in vigorous or moderate PA, for at least ten minutes without interruption^35^. The time of a specific activity is then multiplied with its metabolic equivalent task (MET). The intensity of the activity is reflected in the MET values, whereby more intensive activities have higher MET values. Based on the IPAQ scoring protocol^36^, PA levels were categorized as low, moderate, or high. A total < 600 MET minutes per week score was classified as low, scores between ≥ 600 and < 3000 as moderate, and scores ≥ 3000 as high. If a person goes for a brisk walk 5 times a week, he or she will slightly exceed the limit of 600 METs and be classified as moderate. A three hour moderate-intensity bike ride is equivalent to approximately 3,000 METs, which would put a person into the high activity level group.

### Symptoms of depression and anxiety (outcome)

The PHQ-9 (Patient Health Questionnaire-9) is a self-report screening tool for depression, consisting of nine items. The total score ranges from 0 to 27, with higher scores reflecting greater severity of depressive symptoms. Score values from 0 to 4 indicate the absence of symptoms of depression. A person with values between 5 and 9 has mild symptoms of depression. A score of ≥ 10 represents moderate to severe symptoms of depression and is often used to identify patients with clinical depression (PSD)^37^. The GAD-7 (Generalized Anxiety Disorder-7) is a self-report screening tool for assessing anxiety symptoms consisting of seven items. The total score ranges from 0 to 21, with higher scores indicating greater severity of anxiety symptoms. While a score of 5–9 represents mild symptoms of anxiety, a score values of ≥ 10 are used to identify patients with clinically significant anxiety^38^.

### Clinical data

Routinely collected clinical data provided insights into stroke severity, functional impairment, multimorbidity, mental health conditions, and body mass index (BMI). Multimorbidity was evaluated using a modified version of the Charlson Comorbidity Index (mCCI), which classified patients as multimorbid if more than one additional condition – beyond the stroke itself – from a predefined list of relevant diseases, such as hypertension, coronary heart disease, or diabetes mellitus, was present^39^. Functional impairment following the stroke was measured using the modified Rankin Scale (mRS), which scores from 0 (no symptoms) to 6 (death) and is widely recognized as a reliable tool for assessing disability in stroke patients^40^. Stroke severity was determined with the National Institute of Health Stroke Scale (NIHSS), which quantifies clinical symptoms post-stroke, with higher scores reflecting greater neurological impairment^41^. A mental health disorder diagnosed at any stage of life before stroke was extracted from the medical record (depression, anxiety disorder, or panic attack).

### Statistical analysis

A descriptive analysis of the total sample characteristics stratified by PA level groups was performed. Categorical variables are presented as absolute numbers and relative frequencies, while continuous variables are summarized as mean with standard deviation or median with interquartile range, as appropriate. Tests for differences between variables in the three subgroups of stroke patients based on their level of PA were performed using Pearson’s chi-squared test (categorical variables) and Kruskal-Wallis test (continuous variables), respectively. Multivariable linear regression models were used to investigate the association between pre-stroke PA and post-stroke symptoms of depression and anxiety at 3 and 12 months after discharge. An initial regression analysis was conducted, adjusting for age and sex to account for their potential confounding effects, providing a partially controlled association. A comprehensive model was subsequently developed, incorporating additional covariables to assess the total effect of pre-stroke PA on the mental health of stroke survivors. The covariables were selected according to the directed acyclic graph (DAG) method using the browser-based version of DAGitty (Version 3.1). The selected covariables are age, sex, multimorbidity, mental health disorder, general health status, prior stroke, BMI, smoking and social network. The assumptions for multivariable linear regression analyses were tested using scatterplots to confirm linearity of associations; normal distribution of the residuals and homoscedasticity were checked graphically as well. The variance inflation factor (VIF) was used to test for multicollinearity. Potential leveraging outliers were identified using Cook’s distances and leverage diagnostic plots. In a subgroup analysis, we calculated the same regression models including only more severe stroke cases (mRS ≥ 4). Another sensitivity analysis was conducted to ensure the robustness of the results and comparability to prior studies. Therefore, the comprehensive models were optimized using a two-sided stepwise selection and included stroke severity (NIHSS at admission) and degree of disability (mRS at admission). In addition, the correlation between depressive symptoms and symptoms of anxiety disorder at 3 months after the stroke was analyzed using spearman’s rank correlation. For statistical tests an alpha level of 0.05 was defined. Statistical analyses were conducted using RStudio Version 4.4.1.

## Results

### Sample characteristics

The SCHANA study included a total of 1,790 participants. Of these, 933 participants (52.1%) completed the questionnaire at 3-month follow-up, and 861 participants (48.1%) completed the questionnaire at 12-month follow-up. A complete case analysis was performed. In the comprehensive model, 821 participants (45.9%) were included in the analysis at 3 months and 768 participants (42.9%) at 12 months post-stroke.

Baseline information of the excluded patient sample due to missing follow-up surveys and data was compared with that of analyzed participants to estimate potential selection bias. Excluded participants were older and had lower educational status. The excluded sample had a more balanced gender distribution, with fewer proportion of men (53.0% vs. 61.1%) compared to the analyzed sample. They were also more likely to have experienced a previous stroke and reported significantly lower levels of PA. Furthermore, excluded participants had higher NIHSS and mRS scores, indicating a more severe stroke and greater disability. Higher PHQ-9 scores, indicating a greater severity of depressive symptoms, and lower general health status (EQ-5D VAS score) were also observed in the group of excluded cases. The characteristics of the entire cohort are presented in Table S1 in the supplementary material.

Table 1 provides an overview of the characteristics of the analysis sample at 3 months post-stroke. The analyzed sample population consisted of 502 (61.2%) men and 319 women with a mean age of 68.5 (SD:12.1) years. The majority of participants (*n* = 801, 97.7%) experienced an ischemic stroke and 608 (74.1%) had a first-ever stroke. Multimorbidity was present in almost 70% of all participants. A prior diagnosis of a mental health disorder was documented in 126 (15.3%) of the participants.

**Table 1:** Baseline characteristics of the total sample and stratified by physical activity level group.

Variable	total (*n* = 821)		PA				*p*-value^d^
			low^a^ (*n* = 311, 37.9%)	moderate^b^ (*n* = 284, 34.6%)	high^c^ (*n* = 226, 27.5%)		
Sociodemographic characteristics							
Sex							0.992
Male	502 (61.2)		191 (61.4)	173 (60.9)	138 (61.1)		
Female	319 (38.8)		120 (38.6)	111 (39.1)	88 (38.9)		
Age^e^	68.5 (12.1)		69.7 (12.1)	69.2 (11.6)	66.0 (12.3)		0.001
Education (ISCED-97)							< 0.001
ISCED 1–2	64 (7.8)		35 (11.3)	12 (4.2)	17 (7.5)		
ISCED 3–4	587 (71.5)		229 (73.6)	196 (69.0)	162 (71.7)		
ISCED 5–6	170 (20.7)		47 (15.1)	76 (26.8)	47 (20.8)		
Health-related characteristics							
Type of stroke							0.165
Ischemic stroke	801 (97.7)		303 (97.4)	281 (98.9)	217 (96.0)		
Hemorrhagic stroke	19 (2.3)		8 (2.6)	3 (1.1)	8 (3.5)		
Symptoms of depression (PHQ-9)							
Mean (SD)	5.7 (4.9)		6.5 (5.5)	5.1 (4.4)	5.4 (4.6)		0.014
Median (IQR)	5.0 (2.0;8.0)		5.0 (2.0;9.5)	4.0 (2.0;7.0)	5.0 (2.0;7.8)		
Symptoms of anxiety disorder (GAD-7)							
Mean (SD)	3.9 (4.1)		4.2 (4.5)	3.5 (3.7)	4.2 (4.1)		0.177
Median (IQR)	3.0 (1.0;6.0)		3.0 (1.0;6.0)	3.0 (1.0;5.0)	3.0 (1.0;6.0)		
Prior stroke	213 (25.9)		89 (28.6)	74 (26.1)	50 (22.1)		0.238
Multimorbidity	569 (69.3)		231 (74.3)	192 (67.6)	146 (64.6)		0.042
Mental health disorder	126 (15.3)		51 (16.4)	43 (15.1)	32 (14.2)		0.771
General health status (EQ-5D VAS)^e^	63.4 (20.6)		60.7 (21.0)	65.7 (19.3)	64.4 (21.3)		0.009
BMI^e^	27.6 (5.5)		28.2 (6.1)	27.4 (5.3)	27.1 (5.0)		0.224
Smoking							0.699
Non-smoker	331 (40.3)		124 (39.9)	121 (42.6)	86 (38.1)		
Former smoker	389 (47.4)		145 (46.6)	129 (45.4)	115 (50.9)		
Current smoker	101 (12.3)		42 (13.5)	34 (12.0)	25 (11.1)		
Social network							0.958
Solitary	210 (25.6)		80 (25.7)	71 (25.0)	59 (26.1)		
Cohabiting	611 (74.4)		231 (74.3)	213 (75.0)	167 (73.9)		
NIHSS at admission^f^	1.0 (0.0;3.0)		2.0 (0.0;4.0)	1.0 (0.0;3.0)	2.0 (0.0;3.0)		0.070
mRS at admission^f^	2.0 (1.0;3.0)		2.0 (1.0;3.0)	2.0 (1.0;3.0)	2.0 (1.0;3.0)		0.335

^a^ IPAQ Total Met-minutes/week < 600. ^b^ IPAQ Total Met-minutes/week ≥ 600 to < 3000. ^c^ IPAQ Total Met-minutes/week ≥ 3000. ^d^ Chi-squared test categorical variables; Kruskal-Wallis test for continuous variables. ^e^ Mean (SD). ^f^ Median (Q1; Q3). *BMI = Body-Mass-Index; EQ-5D VAS = EuroQol 5D Questionnaire Visual Analog Scale; GAD = Generalized Anxiety Disorder; MET = Metabolic Equivalent Task; mRS = Modified Rankin Scale; NIHSS = National Institute of Health Stroke Scale; PA = International Physical Activity Questionnaire (IPAQ); PHQ = Patient Health Questionnaire.*

**Fig. 1:**
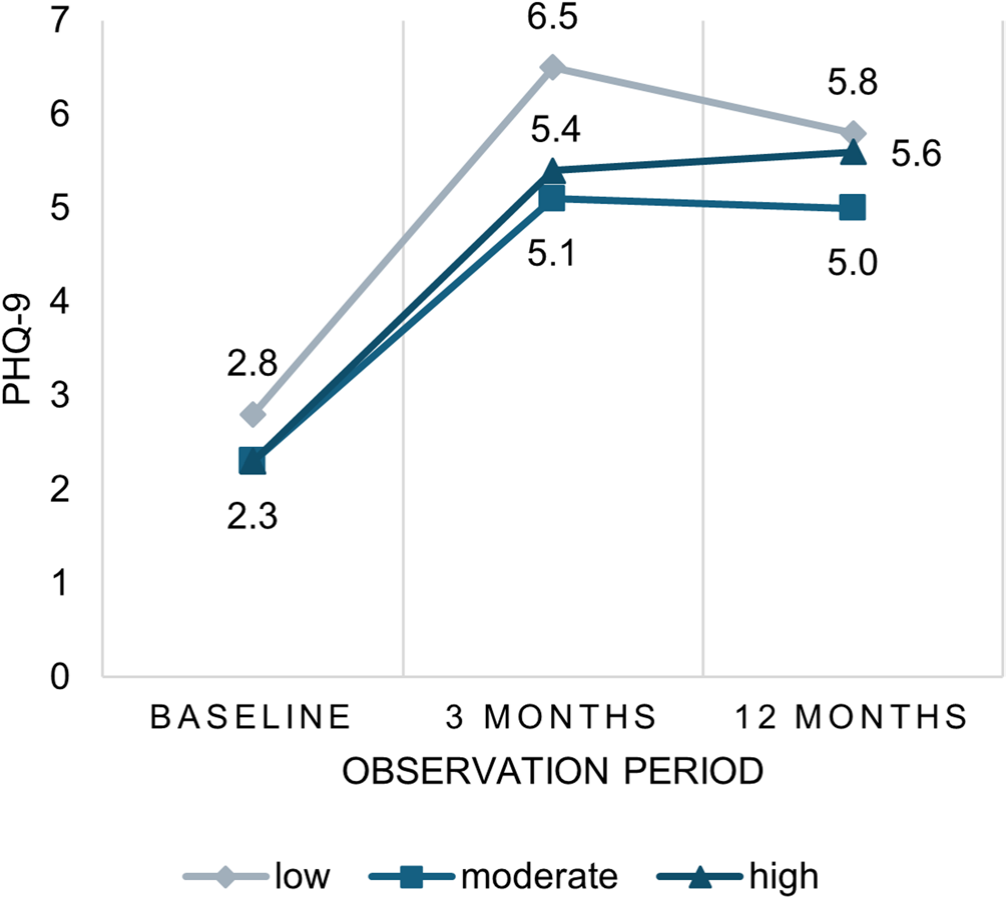
Development of PHQ-9 score by PA level group during the observation period.

Of the 821 participants, 311 (37.9%) had a low level of PA, 284 (34.6%) had a moderate PA level, and 226 (27.5%) had a high PA level. Participants with high PA level were younger compared to participants with low and moderate PA. There was a significant difference in PHQ-9 scores between low and moderate PA groups at 3 months post-stroke (6.5 vs. 5.1), see Fig. 1. The low PA group had a higher proportion of multimorbid participants and reported a poorer general health status compared to moderate and high PA groups.

**Fig. 2:**
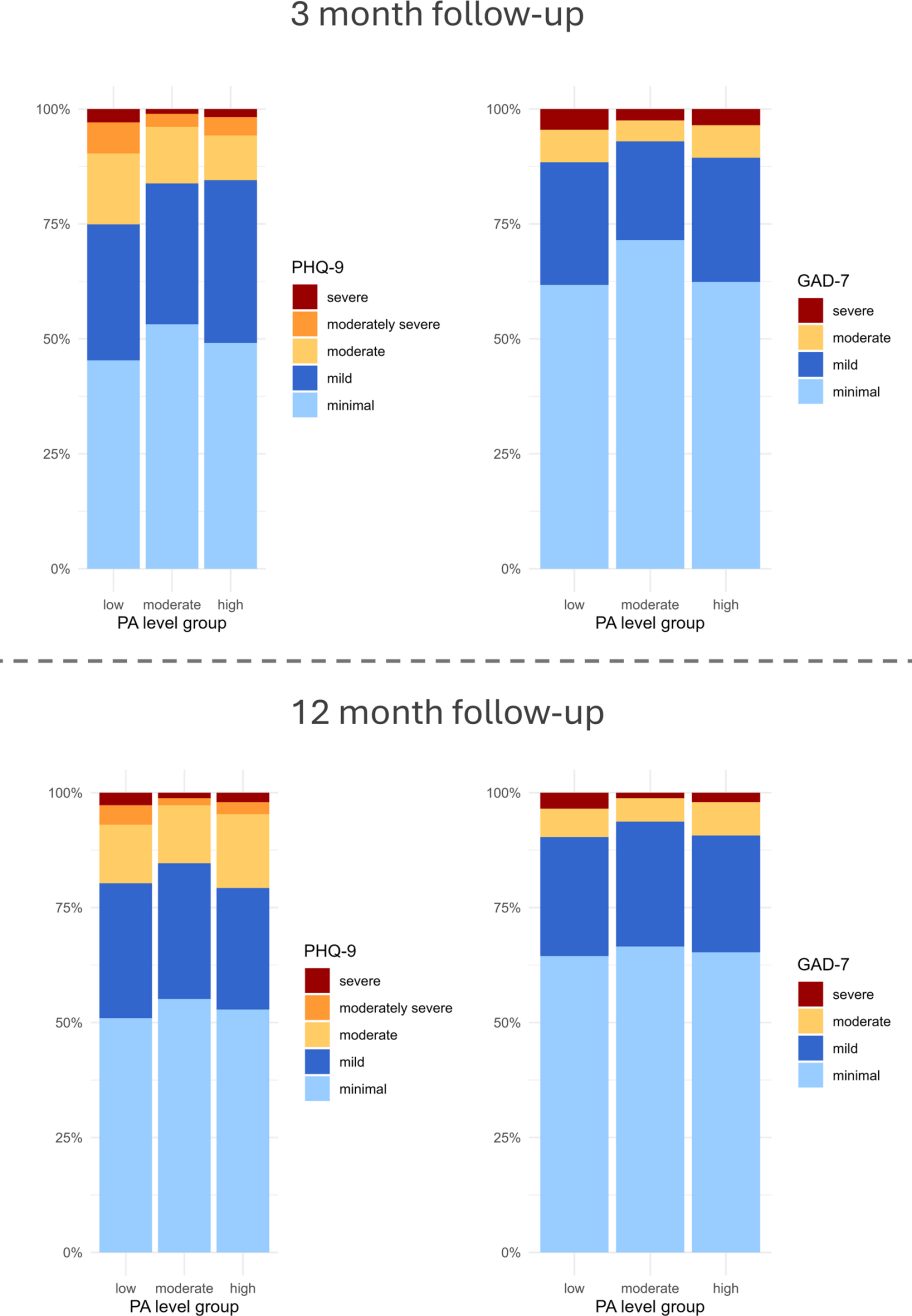
Proportion of severity of depressive symptoms (PHQ-9) and anxiety disorder (GAD-7) between PA level groups at 3-months and 12-month post-stroke. Observations were distributed as follows: low PA (*n* = 311), moderate PA (*n* = 284), and high PA (*n* = 226). Cut-offs for PHQ-9 and GAD-7 score are minimal (0–4), mild (5–9), moderate (10–14), moderately severe (15–19)* and severe (≥ 20 or ≥ 15). *The category “moderately severe” does not exist in the GAD-7 score classification and is therefore not included in this figure.

At 3 months post-stroke, the proportion of participants with PSD (PHQ-9 ≥ 10) was highest in the low PA group at 25.1% (*n* = 78), compared to 16.2% (*n* = 46) in the moderate PA group and 15.5% (*n* = 35) in the high PA group. The proportion of participants with anxiety disorder (GAD-7 ≥ 10) was 11.6% (*n* = 36) in the low PA group, 7.0% (*n* = 20) in the moderate PA group, and 10.6% (*n* = 24) in the high PA group (see Fig. 2). The distribution at the 12-month follow-up was very similar with overall slightly less symptoms of depression and anxiety, see Fig. 2.

### Association between pre-stroke PA and depressive symptoms 3- and 12-months post-stroke

The results of the multivariable linear regression analysis for depressive symptoms at 3- and 12- months post-stroke are shown in Table 2. At 3 months post-stroke, participants with moderate PA had significantly lower PHQ-9 scores compared to those with low PA (reference) in both the partially adjusted model (β = −1.68, *p* < 0.001) and the comprehensive model (β = −1.01, *p* = 0.007). For participants with high PA, the association was significant in the partially adjusted model (β = −1.26, *p* = 0.002) but was not significant in the comprehensive model. At 12 months post-stroke, a significant association with lower PHQ-9 scores was observed for participants with moderate PA in the partially adjusted model (β = −1.02, *p* = 0.010). However, this association was no longer significant in the comprehensive model. For participants with high PA, no significant associations were found one year after discharge, neither in the partially adjusted model nor in the full model. The subgroup analysis including only more severe cases confirmed the main results (similar ß coefficients, but reduced power due to a lower number of included cases), see supplementary table S3. Moreover, the alternative comprehensive regression model, based on a two-sided stepwise selection and additionally including mRS and NIHSS at admission, confirmed the previously shown associations (see Table S2, supplementary material). However, for high PA at 3 months, a significant association with fewer depressive symptoms was observed.

**Table 2:** Multivariable linear regression model analyzing the association between pre-stroke physical activity and symptoms of depression (PHQ-9) 3 and 12 months post-stroke.

Variable	Adjusted *R*^2^	3 months post-stroke			Adjusted *R*^2^	12 months post-stroke		
		Beta	95% CI	*p*-value		Beta	95% CI	*p*-value
*Model 1*	0.021				0.009			
PA moderate		−1.68	−2.42;−0.94	< 0.001***		−1.02	−1.80;−0.24	0.010*
PA high		−1.26	−2.05;−0.47	0.002**		−0.31	−1.15;0.53	0.471
*Model 2*	0.164				0.163			
PA moderate		−1.01	−1.74;−0.28	0.007**		−0.49	−1.25;0.26	0.201
PA high		−0.71	−1.50;0.08	0.077		0.18	−0.64;1.00	0.660
Observations	Model 1		931				861	
	Model 2		821				768	

*Model 1 adjusted by age and sex.*

*Model 2 adjusted by age*,* sex*,* multimorbidity*,* mental health disorder*,* general health status*,* prior stroke*,* BMI*,* smoking and social network.*

*CI = Confidence interval; PA = International Physical Activity Questionnaire; PHQ = Patient Health Questionnaire*

*p < 0.05 | **p < 0.01 | ***p < 0.001

### Association between pre-stroke PA and symptoms of anxiety 3 and 12 months post-stroke

The results of the multivariable linear regression analysis for symptoms of anxiety disorder at 3 and 12 months post-stroke are presented in Table 3. At 3 months post-stroke, participants with moderate PA showed a significant negative association in the partially adjusted model (β = −0.86, *p* = 0.007), while no significant association was observed for high PA compared to low PA group (reference). In the comprehensive model, neither moderate PA nor high PA were significantly associated. At 12-months post-stroke, the associations were not significant in either model for both moderate and high PA level groups. Like for symptoms of depression, the subgroup analysis solely based on more severe stroke cases supported the associations found in the main model, see supplementary table S3. The sensitivity analysis (comprehensive models including the covariables mRS and NIHSS at admission) further confirmed the observed associations (see Table S2, supplementary material).

**Table 3:** Multivariable linear regression model analyzing the association between pre-stroke physical activity and symptoms of anxiety (GAD-7) 3 and 12 months post-stroke.

Variable	Adjusted *R*^2^	3 months post-stroke			Adjusted *R*^2^	12 months post-stroke		
		Beta	95% CI	*p*-value		Beta	95% CI	*p*-value
*Model 3*	0.023				0.013			
PA moderate		−0.86	−1.49;−0.24	0.007**		−0.59	−1.22;0.04	0.065
PA high		−0.31	−0.98; 0.35	0.357		−0.41	−1.09;0.27	0.235
*Model 4*	0.136				0.133			
PA moderate		−0.46	−1.08;0.17	0.151		−0.25	−0.88;0.37	0.427
PA high		0.10	−0.57;0.77	0.770		−0.02	−0.70;0.66	0.953
Observations	Model 3		931				861	
	Model 4		821				768	

Model 3 adjusted by age and sex.

*Model 4 adjusted by age*,* sex*,* multimorbidity*,* mental health disorder*,* general health status*,* prior stroke*,* BMI*,* smoking and social network.*

*CI = Confidence interval; GAD = Generalized Anxiety Disorder; PA = International Physical Activity Questionnaire*

Note: *p < 0.05 | **p < 0.01 | ***p < 0.001

### Correlation between depression and anxiety at 3 months post-stroke

Figure S1 (supplementary material) shows the linear relationship between PHQ-9 and GAD-7 scores at the 3-month follow-up, with a strong and highly significant correlation between symptoms of depression and anxiety (*r* = 0.75, *p* < 0.001). Among patients with PSD (*n* = 159, 19.4%), 42.1% had PSA, while among those with PSA (*n* = 80, 9.7%), 83.8% had PSD after 3 months following an acute stroke event (see Table S4, supplementary material).

## Discussion

Moderate PA before stroke is associated with reduced depressive symptoms within the first three months following the acute stroke event. High pre-stroke PA showed also a protective effect at 3 months post-stroke but only in sensitivity analysis. Pre-stroke PA was not significantly associated with depressive symptoms after 12 months. While no direct link was found between pre-stroke PA and symptoms of anxiety at either follow-up time point, their high correlation with depression suggests that addressing depression through PA before stroke might indirectly influence also anxiety. Notably, participants with lower PA levels before stroke were more likely to report multimorbidity and poorer general health status, potentially exacerbating depressive symptoms.

### Current literature on physical activity and mental health in stroke patients

To this date, there are not many studies that have investigated the association of pre-stroke physical activity and mental health outcomes after stroke. Bovim et al. (2019) and Vestergaard et al. (2023) both highlight the protective effect of pre-stroke PA regarding depressive symptoms during the subacute phase after stroke^42^. Bovim et al. analyzed a smaller sample of 205 patients, while Vestergaard et al. investigated a cohort of 554 participants. Additionally, the follow-up periods differed for both studies: Bovim et al. focused on depressive symptoms 3 months post-stroke, whereas Vestergaard et al. evaluated symptoms at 1 and 6 months. Moreover, different measurement tools were utilized for assessing pre-stroke PA (HUNT2 vs. PASE) and post-stroke depressive symptoms (HADS vs. MDI). While the studies differ in their design and methodology, providing complementary insights into the role of pre-stroke PA on symptoms of PSD, they consistently demonstrate the association between higher pre-stroke PA and reduced depressive symptoms in the subacute phase of stroke recovery. These findings align with the results of the SCHANA study for the moderate PA group. However, our study shows that a high PA level is not significantly associated with a reduction in depressive symptoms compared to the low PA group, indicating a non-linear relationship between physical activity before stroke and mental health in the recovery phase after stroke. A dose-dependent non-linear relationship between physical activity and various health domains is also reported in prior literature^43^. For example intensive exercise might have adverse effects on (cardiovascular) health in professional sports athletes^44^. However, it´s application in stroke patients remains unclear. The results of the Copenhagen City Heart Study indicated that moderate sports activities is associated with the lowest cardiovascular and overall mortality in healthy adults^45^. The results of our study support the idea that more physical activity might not always be beneficial. However, the evidence is clear that very low amounts of physical activity is detrimental to almost all domains of human health and should be avoided in any case^46^.

Prior comparative studies may not represent the total effect of pre-stroke PA due to the adjustment for stroke severity and degree of disability after stroke. In a sensitivity analysis we also adjusted the regression models for those variables, which confirmed this assumption. In our view, however, these variables must be considered mediating factors rather than actual confounders, so they were not included in the main models.

Previous studies showed that the acceptance of post-stroke disability is an important factor in reducing depressive symptoms^47^. Somehow surprisingly, we only found significantly less symptoms of depressive symptoms 3 months after stroke for the moderate PA group, not for the high PA group, however. It could be assumed that highly active patients find it more difficult to adapt to physical and cognitive limitations after stroke, as this represents a drastic shift compared to their previous lifestyle. PA before stroke can be regarded as a potential reflection of an individual’s broader lifestyle. Patients with a high pre-stroke PA level may develop less effective coping strategies to deal with the negative effects of stroke. In contrast, patients with moderate PA might adapt better to the stroke-related limitations.

Depression and anxiety are known to frequently co-occur, as demonstrated in recent studies^6,48^. Our findings add to the existing evidence by showing a clear correlation between higher PHQ-9 and elevated GAD-7 scores. However, pre-stroke PA was primarily associated with a reduction in depressive symptoms and showed no significant effect on anxiety. This aligns with the results observed by Bovim et al. (2019). Nevertheless, given the high correlation between depression and anxiety, it may be plausible that PA exerts an indirect effect on anxiety by mitigating depressive symptoms. However, the causal relationship between depression and anxiety are supposed to be bidirectional, which means that anxiety might also precede depressive symptoms^49^. This potential pathway warrants further investigation to better understand the interconnected psychological outcomes influenced by PA.

### Potential mechanisms of pre-stroke PA impacting post-stroke depression

As prior studies have demonstrated, PA before stroke is associated with protective effects against symptoms of PSD. Regular PA enhances the release of neurotrophic factors, such as Brain-Derived Neurotrophic Factor, which promotes neuronal growth and recovery, supporting functional regeneration^27^. PA also reduces systemic inflammatory markers like C-reactive protein (CRP) and interleukins, and thereby lowering neuroinflammation^27,29^. Furthermore, it maintains the integrity of the blood-brain barrier, reducing the risk of secondary neuroinflammatory damage post-stroke^28^. PA improves vascular health by lowering blood pressure, optimizing lipid metabolism, and enhancing insulin sensitivity, thereby potentially reducing the risk of severe strokes^27,50^. It also stimulates cerebral angiogenesis, improving oxygen supply and functional recovery in the brain^29,50^. These mechanisms may collectively contribute to lower stroke severity and reduced functional impairments, leading to fewer psychological complications in the subacute phase after stroke^10^. However, in the long-term recovery process factors other than pre-stroke physical activity might become more and more influential, which is represented by the circumstance that we found no more significant associations for the 12-month follow-up anymore. Furthermore, as our data indicates, symptoms of depression and anxiety are slightly lower 12 months after stroke compared to the 3-month time point, which makes it more difficult to find significant associations. This is further impeded by a reduced sample size at the 12-month follow-up and consequently less statistical power, but it appears plausible that the effects of pre-stroke physical activity diminish over time and factors like physical activity after stroke are becoming increasingly important in the long run. In the present study, we were not able to analyze the associations between post-stroke physical activity and mental health in the long-term phase of recovery as no information on physical activity after stroke was available.

### Clinical implications and future research

The findings of this study have important implications for clinical practice, particularly for neurologists and primary care physicians. Identifying and addressing depressive symptoms after a stroke should be a priority considering the negative impacts on quality of life and development of an increased risk profile^7–10^. Considering pre-stroke physical activity might be helpful in post-stroke care planning by identifying patients with an increased risk of developing post-stroke mental health issues. A particular attention should be given to those patients who were either physically inactive or highly active prior to stroke. Patients with pre-existing physical limitations are at a heightened risk of developing PSD, highlighting the need for early screening and proactive management of depressive symptoms in this group. Equally, patients who were previously fit and physically active should not be overlooked. The abrupt change in lifestyle and potential loss of autonomy could lead to a lower acceptance of stroke-related impairments. However, also those patients with moderate PA before stroke should be monitored as they might develop PSD later than three months after stroke. Routine mental health assessments during follow-up visits can help detect early signs of PSD in these individuals. While an active lifestyle before stroke might offer protective effects against depressive symptoms through neurovascular mechanisms, these benefits may be mitigated by a lack of acceptance of stroke-related disability. However, we cannot draw conclusion about causality of the associations described and consequently we would be careful to derive concrete recommendations for patients based on results of this study. Especially the non-linear relationship between physical activity and mental health after stroke indicated by our results makes it difficult to give general recommendations. Moreover, there might be a relevant uncertainty or overestimation of the actual amount of physical activity based on self-reported information given by the patients^51^, which makes it not easier to give general advise to patients based on results from the present study. With these limitations in mind, we still consider PA to be valuable for screening and identifying patients with increased risk of depression or anxiety after stroke. Future research should investigate how pre-stroke PA levels influence the acceptance of stroke-related disability and the interplay between depression and anxiety in stroke survivors.

Our findings underline the need for psychological care for seriously ill patients (e.g., stroke patients), which aligns with the objectives of the ongoing digiBRAVE project in Augsburg, Germany^52^. This Bavarian State Ministry-funded initiative aims to utilize modern digital tools to predict, prevent, and improve the treatment of depressive episodes in patients with severe somatic diseases, including stroke. By employing a stepped-care model, digiBRAVE offers tailored digital interventions that adapt to the severity and individual preferences of patients, trying to promote a comprehensive and innovative approach to mental health care.

### Strengths and limitations

Our study is characterized by several strengths. The large sample size provides sufficient statistical power, and the longitudinal design allows valuable insights into changes over time. Reliable data was collected by standardized personal interviews and elaborating medical records. Moreover, validated instruments were used to measure mental health outcomes^38,53^. Adjustment for potential confounders approximates a causal effect, and sensitivity analyses reinforced the robustness of our findings.

However, there are also some limitations to mention. First, as an observational study, it cannot establish causality between pre-stroke PA and mental health outcomes after stroke. However, the prospective design and adjustment for potential confounders strengthen the observed associations. Second, there might be a potential selection bias, as the sample consists mainly of patients with minor strokes and mild disabilities, which may limit generalizability to more severe cases. However, the subgroup analyses indicated that the reported associations are also valid for more severe cases (mRS ≥ 4).Third, even though the IPAQ is a validated tool widely used in similar studies^35^, a limitation with IPAQ is recall bias, which is confirmed by studies like Lee et al., who found that self-assessed PA often overestimates objective measures.^51^.This might especially affect the found associations for the high physical activity group. Also, information on physical activity was only available at baseline. Stroke-related impairments can affect a patient’s ability to engage in physical activity after the event, so examining the association between post-stroke physical activity and mental health outcomes might help to clarify the associations and to get a more comprehensive understanding of the underlying interconnection. Additionally, conducting the study partly during the COVID-19 pandemic may limit generalizability due to pandemic-related behavioral changes. Lastly, we did not assess antidepressant use, which could influence depression outcomes. However, Vestergaard et al. (2023) found that antidepressant use did not significantly affect the association between pre-stroke PA and depression.

## Conclusion

Moderate PA before stroke is associated with lower levels of depressive symptoms 3 months after stroke but not 12 month post-stroke. Notably, higher levels of PA before stroke are not associated with greater reductions in depressive symptoms. The high correlation between PSD and PSA suggests that addressing depression through PA before stroke may indirectly influence anxiety outcomes. Regarding mental well-being, physicians should pay particular attention to stroke patients who were either physically inactive or highly active prior to stroke.

## Supplementary Information

Below is the link to the electronic supplementary material.

Supplementary Material 1

## Data Availability

The raw data supporting the conclusions of this article will be made available by the authors upon reasonable request.
