# Supplementary material for: Moderate pre-stroke physical activity has a protective effect on symptoms of depression in the post-acute phase after stroke
Source: Sci Rep. 2026 May 26;16:16290. doi: 10.1038/s41598-026-51679-5 (PMC13212948; doi:10.1038/s41598-026-51679-5)
Supplement: Supplementary file 1 — Supplementary Material 1 [file 41598_2026_51679_MOESM1_ESM.docx]

# ***Supplementary Material***

# **Title:** Moderate pre-stroke physical activity has a protective effect on symptoms of depression in the post-acute phase after stroke.

# Moritz Hahner, Christine Meisinger, Inge Kirchberger, Michael Ertl, Markus Naumann, Jakob Linseisen, Timo Schmitz

# **Supplementary Table S1** | Comparison of analyzed and excluded patient samples at baseline (n, %)

| **Variable** | **analyzed (n = 821)** | **excluded**^a^ **(n = 969)** | **p-value**^b^  **-** | **missing values (excluded)** |
| --- | --- | --- | --- | --- |
| **Sociodemographic characteristics** |  |  |  |  |
| Sex | 821 (100) | 953 (98.3) | 0.003 | 16 |
| Male | 502 (61.1) | 514 (53.0) |  |  |
| Female | 319 (38.9) | 439 (45.3) |  |  |
| Age^c^ | 68.5 (12.1) | 69.8 (14.0) | 0.002 | 11 |
| Education (ISCED-97) | 821 (100) | 682 (70.4) | <0.001 | 287 |
| ISCED 1-2 | 64 (7.8) | 99 (10.2) |  |  |
| ISCED 3-4 | 587 (71.5) | 487 (50.3) |  |  |
| ISCED 5-6 | 170 (20.7) | 96 (9.9) |  |  |
| **Health-related characteristics** |  |  |  |  |
| Typ of stroke | 820 (99.9) | 965 (99.6) |  | 4 |
| Ischemic stroke | 801 (97.6) | 929 (95.9) | 0.072 |  |
| Hemorrhagic stroke | 19 (2.3) | 36 (3.7) | 0.114 |  |
| Prior stroke | 213 (25.9) | 248 (25.6) | 0.001 | 229 |
| Physical activity  (IPAQ Total-Met minutes/week) |  |  | <0.001 | 250 |
| Mean (SD) | 2205.6 (2733.4) | 1347.0 (2558.1) |  |  |
| Median (IQR) | 1188 (297;3333) | 0 (0;1440) |  |  |
| Multimorbidity | 569 (69.3) | 702 (72.4) | 0.160 |  |
| Mental health disorder | 126 (15.3) | 121 (12.5) | 0.215 | 292 |
| General health status (EQ-5D VAS)^c^ | 63.4 (20.6) | 58.5 (22.1) | <0.001 | 291 |
| BMI^c^ | 27.6 (5.5) | 27.0 (5.4) | 0.030 | 125 |
| Smoking | 821 (100) | 969 (100) | <0.001 |  |
| Non-smoker | 331 (40.3) | 567 (58.5) |  |  |
| Former smoker | 389 (47.4) | 282 (29.1) |  |  |
| Current smoker | 101 (12.3) | 120 (12.4) |  |  |
| Social network | 821 (100) | 661 (68.2) | <0.001 | 308 |
| Solitary | 210 (25.6) | 228 (23.5) |  |  |
| Cohabiting | 611 (74.4) | 433 (44.7) |  |  |
| NIHSS at admission^d^ | 1.0 (0.0;3.0) | 2.0 (0.0;5.0) | <0.001 | 76 |
| mRS at admission^d^ | 2.0 (1.0;3.0) | 3.0 (1.0;4.0) | <0.001 | 72 |
| Symptoms of depression (PHQ-9)^c,d^ |  |  | <0.001 | 328 |
| Mean (SD) | 2.5 (3.4) | 3.3 (4.1) |  |  |
| Median (IQR) | 1.0 (0.0;4.0) | 2.0 (0.0;5.0) |  |  |
| ^a^ Only patients with available data are reported ^b^ Chi-squared test for categorical variables; Kruskal-Wallis test for continuous variables ^c^ Mean (SD) ^d^ Median (Q1; Q3)  *BMI = Body-Mass-Index; EQ-5D VAS = EuroQol 5D Questionnaire Visual Analog Scale; IPAQ = International Physical Activity Questionnaire; ISCED-97 = International Standard Classification of Education 1997; MET = Metabolic Equivalent Task; mRS = Modified Rankin Scale; NIHSS = National Institute of Health Stroke Scale; PA = International Physical Activity Questionnaire; PHQ = Patient Health Questionnaire* | | | | |

**Supplementary Table S2 |** Sensitivity analysis, multivariable linear regression models analyzing the association between pre-stroke PA and depression (PHQ-9) and anxiety (GAD-7) at 3 and 12 months post-stroke

|  |  | **3 months post-stroke** | | |  | **12 months post-stroke** | | |
| --- | --- | --- | --- | --- | --- | --- | --- | --- |
| **Variable** | **Adjusted R^2^** | **Beta** | **95% CI** | **p-value** | **Adjusted R^2^** | **Beta** | **95% CI** | **p-value** |
| *PHQ-9* | 0.176 |  |  |  | 0.174 |  |  |  |
| PA moderate | | -1.05 | -1.79;-0.30 | 0.006** |  | -0.52 | -1.29;0.26 | 0.192 |
| PA high |  | -0.86 | -1.65;-0.07 | 0.033* |  | 0.01 | -0.82;0.84 | 0.983 |
| *GAD-7* | 0.131 |  |  |  | 0.119 |  |  |  |
| PA moderate | | -0.61 | -1.26;0.03 | 0.061 |  | -0.40 | -1.05;0.25 | 0.230 |
| PA high |  | -0.06 | -0.75;0.62 | 0.854 |  | -0.16 | -0.86;0.54 | 0.661 |
| Observations | |  | 787 |  |  |  | 731 |  |
| *Models adjusted by age, sex, multimorbidity, mental health disorder, general health status, prior stroke, BMI, mRS at admission and NIHSS at admission.*  *CI = Confidence interval; GAD = Generalized Anxiety Disorder; mRS = Modified Rankin Scale; NIHSS = National Institute of Health Stroke Scale; PA = International Physical Activity Questionnaire; PHQ = Patient Health Questionnaire*  *Note: *p<0.05 \| **p<0.01 \| ***p<0.001* | | | | | | | | |

**Supplementary Table S3 |** Subgroup analysis including only more severe cases (mRS at admission ≥4). Multivariable linear regression models were used to analyze the association between pre-stroke PA and depression (PHQ-9) and anxiety (GAD-7) at 3 and 12 months post-stroke

|  |  | **3 months post-stroke** | | |  | **12 months post-stroke** | | |
| --- | --- | --- | --- | --- | --- | --- | --- | --- |
| **Variable** | **Adjusted R^2^** | **Beta** | **95% CI** | **p-value** | **Adjusted R^2^** | **Beta** | **95% CI** | **p-value** |
| *PHQ-9* | 0.161 |  |  |  | 0.163 |  |  |  |
| PA moderate | | -1. 50 | -2.28;-0.17 | 0.028* |  | -0.49 | -1.25;0.26 | 0.201 |
| PA high |  | -1.14 | -2.48;-0.20 | 0.095 | 0.18 | -0.64;1.00 | 0.660 | 0.983 |
| *GAD-7* | 0.113 |  |  |  | 0.133 |  |  |  |
| PA moderate | | -1.22 | -2.33;-0.12 | 0.030* |  | -0.25 | -0.88;0.37 | 0.427 |
| PA high |  | 0.15 | -0.95; 1.25 | 0.790 |  | -0.02 | -0.70;0.66 | 0.953 |
| Observations | |  | 787 |  |  |  | 731 |  |
| *Models adjusted by age, sex, multimorbidity, mental health disorder, general health status, prior stroke, BMI, smoking and social network.*  *CI = Confidence interval; GAD = Generalized Anxiety Disorder; mRS = Modified Rankin Scale, PA = International Physical Activity Questionnaire; PHQ = Patient Health Questionnaire*  *Note: *p<0.05 \| **p<0.01 \| ***p<0.001* | | | | | | | | |

**Table S4 |** Co-occurrence of depression and anxiety disorder at 3 months post-stroke

|  | **GAD-7** | |  |
| --- | --- | --- | --- |
| **PHQ-9** | **No Anxiety Disorder** | **Anxiety Disorder**^a^ | **total** |
| **No Depression** | 649 | 13 | 662 |
| **Depression**^b^ | 92 | 67 | 159 |
| **total** | 741 | 80 | 821 |
| ^a^ GAD-7 score ≥10 ^b^ PHQ-9 score ≥10  *GAD = Generalized Anxiety Disorder; PHQ = Patient Health Questionnaire* | | | |


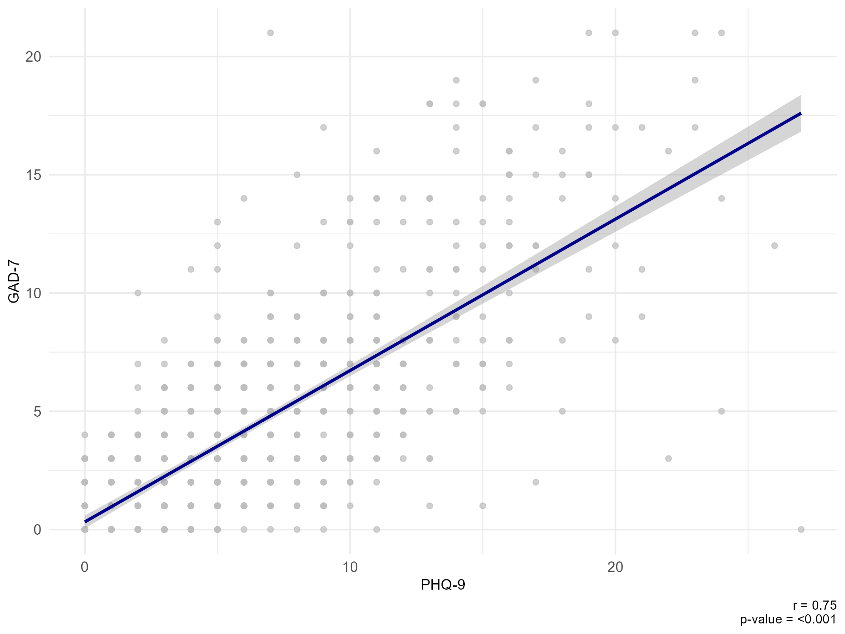


**Figure S1** **|** Correlation between depression and anxiety at 3 months post-stroke
